# Motion and Form Perception in Childhood-Onset Schizophrenia

**DOI:** 10.3390/pediatric16010009

**Published:** 2024-01-15

**Authors:** Szabolcs Kéri, Oguz Kelemen

**Affiliations:** 1 Sztárai Institute, University of Tokaj, 3944 Sárospatak, Hungary; 2 Department of Physiology, Albert Szent-Györgyi Medical School, University of Szeged, 6720 Szeged, Hungary; 3 Department of Behavioral Science, Albert Szent Györgyi Medical School, University of Szeged, 6720 Szeged, Hungary; kelemen.oguz@med.u-szeged.hu; 4 Department of Psychiatry, Bács-Kiskun County Hospital, 6000 Kecskemét, Hungary

**Keywords:** childhood-onset schizophrenia, neurodevelopment, dorsal visual stream, motion perception, cognition

## Abstract

(1) Background: Childhood-onset schizophrenia (COS) is a rare type of psychotic disorder characterized by delusions, hallucinations, grossly disorganized behavior, and poor psychosocial functioning. The etiology of COS is unknown, but neurodevelopmental factors are likely to play a critical role. A potential neurodevelopmental anomaly marker is the dorsal visual system dysfunction, which is implicated in motion perception, spatial functions, and attention. (2) Methods: To elucidate the role of the dorsal visual system in COS, we investigated 21 patients with COS and 21 control participants matched for age, sex, education, IQ, and parental socioeconomic status. Participants completed a motion and form coherence task, during which one assesses an individual’s ability to detect the direction of motion within a field of moving elements or dots and to recognize a meaningful form or object from a set of fragmented or disconnected visual elements, respectively. (3) Results: The patients with COS were impaired in both visual tasks compared to the control participants, but the evidence for the deficit was more substantial for motion perception than for form perception (form: BF_10_ = 27.22; motion: BF_10_ = 6.97 × 10^6^). (4) Conclusions: These results highlight the importance of dorsal visual stream vulnerability in COS, a potential marker of neurodevelopmental anomalies.

## 1. Introduction

Childhood-onset schizophrenia (COS) is a rare (prevalence: 1 in 40,000) and severe mental disorder that affects children before the age of 13 years [1,2]. It is a complex condition characterized by a range of symptoms, including hallucinations, delusions, disorganized thinking, and social withdrawal [3]. It presents a unique set of challenges due to its early onset, making it distinct from adult-onset schizophrenia. COS poses diagnostic challenges due to the difficulty of distinguishing between normal childhood behaviors and symptoms of the disorder [4,5]. 

Children with COS often experience cognitive impairments, affecting their ability to think clearly, concentrate, and learn. This can lead to academic difficulties and hinder their overall intellectual development. Additionally, social withdrawal and impaired social interactions are common in COS. Children may struggle to form and maintain relationships, leading to feelings of isolation and loneliness [6,7]. 

The exact causes of COS remain unknown, but research suggests a combination of genetic, neurobiological, and environmental factors [8,9,10,11]. Genetic predisposition plays a significant role, as children with a family history of schizophrenia are at a higher risk. Neurobiological factors, such as abnormal brain development and neurotransmitter imbalances affecting glutamatergic, GABA-ergic (gamma-amino-butyric acidergic), and dopaminergic synapses, are also believed to contribute to the onset of COS [8,11]. Furthermore, environmental factors, including prenatal complications, maternal stress, and childhood trauma, may increase the risk of developing the disorder [4,12]. 

Studies suggest that even schizophrenia appearing in adolescence and early adulthood is linked to irregular growth and maturation of the brain before and during childhood [13,14,15,16]. High-risk studies revealed that the cognitive, language, motor, social, and emotional development of children may be impaired before the emergence of schizophrenia [17,18,19]. In addition, these dysfunctions profoundly affect the lives of children. Multiple areas of behavior and cognition are impaired in COS. Patients with COS often experience cognitive deficits, including difficulties with attention, memory, problem solving, and executive functions [20,21]. These impairments can impact their academic performance and overall cognitive development. Children with schizophrenia and even individuals with an increased risk may struggle with social interactions and exhibit impaired social skills [22,23,24]. 

Children with schizophrenia often experience delays or impairments in language development. They may have difficulties with expressive language, comprehension, and verbal fluency. These language and communication problems can further hinder their social interactions and academic progress [25,26]. Motor abnormalities are commonly observed in children with schizophrenia. These can include motor coordination difficulties, abnormal movements, and unusual postures. These motor alterations can impact their daily activities and overall motor development [24,27,28]. 

Some children with schizophrenia may exhibit aggression, impulsivity, and self-harm. These behavioral issues can be a result of their cognitive impairments, social difficulties, and the distress caused by their psychotic symptoms [29]. Overall, children with schizophrenia often experience a decline in academic performance compared to their peers [30], which is partly due to various comorbid conditions (e.g., specific learning disorders, disruptive behavioral disorders, disruptive mood dysregulation disorder, attention-deficit hyperactivity disorder, and epilepsy) [31]. This decline can be attributed to their cognitive impairments, difficulties with attention, and disrupted learning due to psychotic symptoms. Early identification, intervention, and ongoing support are crucial in addressing these developmental alterations and promoting the overall well-being of children with schizophrenia. 

With the ambition of recognizing susceptibility and adjusting genes and their connections with environmental elements, one of the primary objectives of the current research is to discover precise endophenotypes, which are measurable and heritable characteristics or traits that are associated with a particular disorder or condition. Unlike the clinical symptoms or manifestations of a disorder, endophenotypes are considered more specific and closer to the underlying genetic and biological mechanisms of the condition, although this concept has not received uniform acceptance in the scientific community [32]. Adding a cognitive evaluation to classic high-risk epidemiological studies is a promising but controversial option [33,34,35]. Analyzing motion and form perception presents a rare chance to acquire a deeper insight into developmental issues, an uninvestigated area in COS. Motion and form perception abnormalities are common in individuals with schizophrenia [36,37,38], but there are exceptions [39]. These perceptual disturbances contribute to the overall cognitive impairments experienced by individuals with the disorder. In terms of motion perception, individuals with schizophrenia often have difficulties perceiving and processing visual motion. They may struggle to accurately perceive the direction, speed, and coherence of moving objects [37,40]. This can lead to problems with tasks that require tracking moving objects or judging the movement of objects in their environment. For example, individuals with schizophrenia may have difficulty crossing a busy street safely or playing sports that involve tracking a moving ball.

Form perception abnormalities in schizophrenia involve difficulties in perceiving and recognizing shapes, patterns, and objects [41,42]. Individuals with the disorder may struggle with tasks that require visual organization and integration, such as identifying faces or objects in complex visual scenes [43,44]. They may also have difficulties with optical illusions, perceiving ambiguous figures, or discriminating between similar shapes or patterns [45]. These motion and form perception abnormalities in schizophrenia are thought to be related to underlying neurobiological and cognitive impairments. Dysfunction in the brain regions that are involved in visual processing, such as the occipital, temporal, and parietal lobes, may contribute to these perceptual disturbances [46,47,48]. In a simplified manner, the ventral visual stream (occipital and inferior temporal regions) is implicated in form perception and object recognition. In contrast, the dorsal visual stream (occipital and parietal—middle temporal cortex) is essential to the processing of spatial location and motion, although object representation also occurs in the dorsal system because of the close interaction between the two streams [49,50,51]. Additionally, deficits in attention, working memory, and executive functioning can further impact the ability to perceive and interpret visual information accurately [52,53]. Understanding these perceptual abnormalities in schizophrenia is essential for both diagnosis and treatment. Assessing motion and form perception can provide valuable insights into the cognitive impairments that are experienced by individuals with the disorder. Additionally, interventions targeting these perceptual disturbances, such as visual training programs or cognitive remediation therapies, may help improve overall cognitive functioning and quality of life for individuals with schizophrenia [54].

Investigations have shown that developmental disorders of the brain, including Williams syndrome [55], autism spectrum disorders [56,57], childhood hemiplegia [58], fragile X syndrome [59], and developmental coordination disorder [60], are distinguished by a deficiency of directional motion processing in comparison to global form processing. The dorsal stream vulnerability idea proposes that motion-sensitive V3a (accessory visual area 3) and V5 (visual area 5)/MT (middle temporal) areas in the dorsal occipito-parietal and middle temporal visual system are especially exposed to genetic and environmental elements that can influence brain maturation and progression [61,62,63]. Ample studies collectively suggest that there is a valid concept called dorsal stream vulnerability in neurodevelopmental disorders, although this concept is still hypothetical. Atkinson (2017) [64] proposed that dorsal stream vulnerability is a cluster of problems related to motion sensitivity, visuomotor spatial integration, attention, and numeric skills and shared across various disorders. Gunn et al. (2002) [58] were among the first to support this idea by finding that children with hemiplegic cerebral palsy perform worse on motion coherence tasks, indicating dorsal stream impairment. Braddick and Atkinson (2013) [65] further discussed the role of the dorsal stream in the visual control of manual actions and suggested that its vulnerability may be a widespread feature of neurodevelopmental disorders. An enigmatic example is Williams syndrome (deletion of 26–28 genes on chromosome 7, resulting in cardiovascular disease, developmental delays, and learning difficulties, but exaggerated verbal abilities and sociability), in which researchers highlighted the pathology of the dorsal stream in relation to spatial cognition, attention, and planning of actions [64]. Overall, these studies provide evidence of dorsal stream vulnerability in neurodevelopmental disorders.

Despite the promising nature of form and motion perception as a proxy marker of neurodevelopmental alterations, these visual measures have not been utilized in COS. The present study aimed to investigate motion and form perception in COS, which is a missing link in the literature. We hypothesized that motion perception is impaired in COS, a hallmark of neurodevelopmental alterations.

## 2. Materials and Methods

### 2.1. Participants and Assessment

We enrolled 21 patients with COS from the psychiatric centers of Hungary, and 21 healthy control children matched for age, sex, education, parental socioeconomic status, and IQ (Table 1).

The diagnosis was based on the medical records of the patients, interviews with the parents, and the Structured Clinical Interview for DSM-5 Disorders—Clinician Version (SCID-5-CV) [66]. SCID-5-CV is a widely used diagnostic tool in mental health for conducting structured interviews to assess and diagnose psychiatric disorders based on the criteria outlined in the *Diagnostic and Statistical Manual of Mental Disorders, Fifth Edition* (DSM-5). The SCID-5-CV is designed to be administered by a trained clinician or researcher and provides a systematic and standardized approach to gathering information about an individual’s symptoms, history, and functioning. It covers a wide range of psychiatric disorders, including mood disorders, anxiety disorders, psychotic disorders, and substance use disorders. The interview consists of questions and prompts that guide the clinician through the diagnostic criteria for each disorder. The clinician assesses the presence and severity of symptoms, duration, and impairment to functioning to determine if the criteria for a specific disorder are met. The SCID-5-CV also includes probes and follow-up questions to gather additional information and clarify responses. The structured nature of the SCID-5-CV helps ensure consistency and reliability in the diagnostic process, reducing subjectivity and increasing the accuracy of diagnoses [65].

We used the Positive and Negative Syndrome Scale (PANSS) to assess the positive symptoms (e.g., hallucinations, delusions, and conceptual disorganization; 7 items, minimum score: 7, maximum score: 49), negative symptoms (e.g., blunted affect, emotional withdrawal, and passive/apathetic social withdrawal; 7 items, minimum score: 7, maximum score: 49), and general symptoms (e.g., somatic concerns, anxiety, guilt feeling, and depression; 16 items, minimum score: 16, maximum score: 112) [67].

The parents’ socioeconomic status of COS patients was assessed with the Hollingshead Four Factor Index [68]. We used the Wechsler Adult Intelligence Scale-IV (WAIS-IV) to evaluate general intellectual functions [69]. The clinical rating scales and interviews were administered by qualified raters who were blind to the aim of the study.

### 2.2. Motion and Form Perception

We applied motion and form coherence measurements. These methods assess an individual’s ability to perceive and integrate visual information that is related to motion and form. These measurements are based on specific principles and tasks, designed to evaluate the coherence of visual stimuli. In motion coherence measurements, a display of randomly moving dots is presented, with a certain percentage of dots moving in a coherent direction. The participant’s task is to identify the direction of the coherent motion. The coherence level (percentage of dots moving coherently) is varied to determine the threshold at which the participant can detect the coherent motion.

In contrast, in form coherence measurements, the task involves stimuli with varying levels of coherence in shape or structure. For example, participants may be presented with fragmented or degraded images and asked to identify the object or shape represented. The coherence level refers to the clarity or completeness of the form or shape [70].

**Figure 1:**
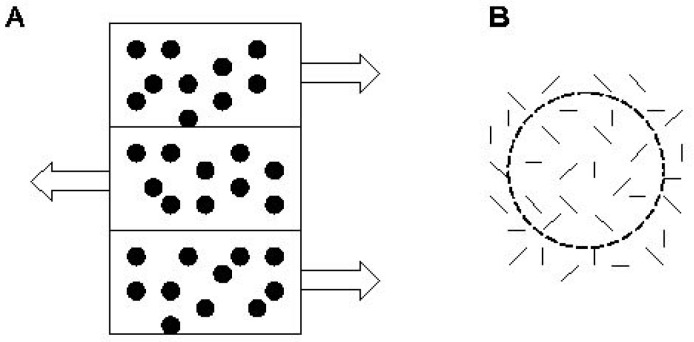
Illustration of the motion perception (**A**) and form perception (**B**) tasks. In panel (**A**), arrows indicate the direction of motion.

Stimuli appeared on a ViewSonic PF815 monitor. In the motion coherence task, stimuli consisted of an array of dots (density = 4 dots/degree^2^, stimulus area luminance = 85 cd/m^2^). Signal dots moved horizontally at 6 degrees/s and reversed direction every 0.4 s. Noise dots were repositioned randomly on each frame. The task was to locate a 12° × 7° strip that appeared either on the screen’s left or right side. In the target strip, the coherently moving signal dots oscillated in the opposite phase in the surrounding regions (Figure 1). Participants made decisions by pressing different keys on the computer keyboard (1 for left and 9 for right). Coherence was the ratio of signal dots to noise dots. At the beginning of the test, each dot (100%) oscillated coherently. This proportion was reduced until the first error when a 2-up/1-down staircase was introduced. The threshold was defined as the average of six reversals.

In the form coherence task, stimuli consisted of 0.4°-long line segments (density = 19 segments/degree^2^). In a circular area with a radius of 7°, localized either to the left or to the right of the center of the screen, some of the line segments were tangentially oriented to form a circle. The surrounding noise line segments were randomly positioned. The proportion of tangentially oriented segments defined the level of coherence (Figure 1). Participants were asked to decide whether the circle appeared on the screen’s left or right side.

In both tasks, the level of coherence at the detection threshold characterizes the visual system’s sensitivity. If a higher level of coherence is necessary for detecting a stimulus (a higher proportion of dots oscillates coherently or a higher proportion of line segments is oriented tangentially), the threshold is higher, and the sensitivity of the visual system is lower [38,71].

### 2.3. Data Analysis

We used STATISTICA 13.1 (Tibco) for data analysis. Following the descriptive statistics, including Kolmogorov–Smirnov to check the normality of the data distribution and Levene’s test to assess the homogeneity of variance, we used analysis of variance (ANOVA) on the coherence thresholds. The between-subjects factor was the study group (COS vs. control participants), whereas the within-subjects factor was task type (motion vs. form coherence). The effect size values (η^2^) were also calculated. Tukey’s Honestly Significant Difference Tests (HSD) were used for post hoc comparisons. We calculated Pearson’s product–moment correlation coefficients between threshold values and clinical symptoms. The level of significance was α < 0.05. The critical between-group differences were confirmed with Bayesian statistics with the JASP 0.17.1 package (Bayes Factor levels of evidence: BF_10_ 1–3: weak evidence, 3–10: moderate evidence, >10: strong evidence).

## 3. Results

### 3.1. Differences between COS and Controls in Motion and Form Perception

**Figure 2:**
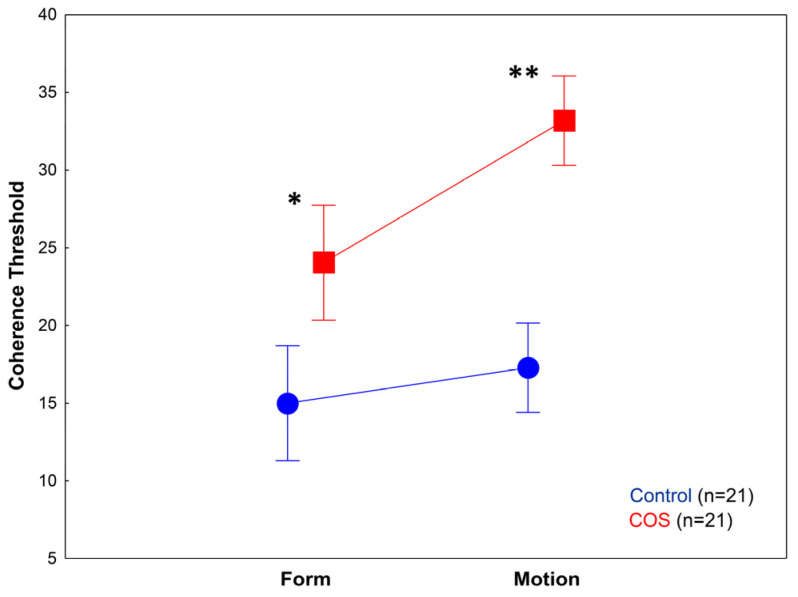
Results from the form and motion perception tasks. Patients with childhood-onset schizophrenia (COS) showed significantly higher coherence threshold values in both conditions, but it was more pronounced in the motion perception task. * *p* = 0.001; ** *p* = 0.0001.

**Figure 3:**
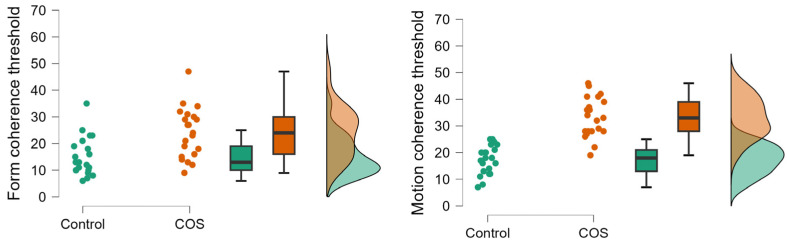
Raincloud plots showing raw data in controls and childhood-onset schizophrenia (COS), mean (vertical line), interquartile range (box plots), 95% confidence intervals (error bars), and density functions.

The two-way ANOVA conducted on the motion and form threshold values revealed significant main effects of group (COS vs. controls) (F(1,40) = 45.59, *p* < 0.001; η^2^ = 0.53) and task type (motion vs. form) (F(1,40) = 16.65, *p* < 0.001; η^2^ = 0.29). The two-way interaction between group and task type was also significant (F(1,40) = 6.0, *p* < 0.05; η^2^ = 0.13). Tukey’s HSD revealed that patients with COS were impaired in both motion (*p* = 0.0001) and form perception tasks (*p* = 0.001) (Figure 2 and Figure 3).

However, motion perception was significantly more challenging for patients with COS than for the comparison group. In the control group, there was no significant difference between the form and motion perception threshold (*p* = 0.66), whereas in the COS group, we observed a significantly higher threshold in the motion task relative to the form task (*p* < 0.05) (Figure 2 and Figure 3).

### 3.2. Correlations between Visual Perception and Clinical Measures

There were no significant correlations between the form and motion threshold values, PANSS scores, IQ, illness duration, or the daily antipsychotic dose (−0.2 < r < 0.2; *p* > 0.1).

### 3.3. Bayesian Statistical Analysis

In order to obtain the strength of evidence for differences between COS and controls, we applied Bayesian *t*-tests to compare the form and motion coherence threshold. Bayesian statistics revealed substantial evidence for the finding that individuals with COS were impaired on the form perception task (BF_10_ = 27.22, error %: 7.45 × 10^−7^). In the motion perception task, the Bayesian level of evidence was extremely strong for impaired performances in COS (BF_10_ = 6.97 × 10^6^, error %: 7.40 × 10^−14^).

## 4. Discussion

We have demonstrated impaired form and motion perception in COS. However, COS-associated motion perception deficits were more pronounced relative to form perception dysfunctions, as revealed by the two-way interaction in the ANOVA and the level of Bayesian evidence. Notably, this deficit was not due to a generalized cognitive deficit, because the COS and control groups were matched for IQ, and there was no significant correlation between the visual coherence threshold values and IQ. Similarly, form and motion perception did not correlate with the severity of COS’s positive, negative, or general symptoms, and it was not the consequence of a longer illness duration. It must be noted, however, that the lack of correlations can be due to the small sample size. Altogether, these results indicate that form and motion perception impairments are not domain-general in COS; they may represent a unique trait marker of the illness, not directly influenced by the fluctuation of clinical symptoms (state markers). While we have demonstrated specific findings about motion detection and form perception in COS, to be able to make a statement that we identified an endophenotype would require characterizing our protocol in multiple childhood disorders.

How can we explain that motion perception is more impaired than form perception in COS? The difference in impairment between motion perception and form perception can be attributed to the distinct neural mechanisms and brain regions that are involved in processing these visual attributes. Motion perception refers to the ability to perceive and interpret the movement of objects or visual stimuli in the environment. It detects and analyzes motion-related cues, such as direction, speed, and trajectory. On the other hand, form perception refers to the ability to perceive and recognize the shape, structure, and boundaries of objects or visual stimuli [72,73]. One possible explanation for the more significant impairment of motion perception compared to form perception is the specialization of brain regions for processing these visual attributes. Research has shown that motion perception primarily relies on the dorsal visual pathway, also known as the “where” pathway, which includes brain areas such as the middle temporal area (MT) and the medial superior temporal area (MST). These regions are responsible for analyzing motion-related information and integrating it with spatial and temporal cues. In contrast, form perception primarily relies on the ventral visual pathway, also known as the “what” pathway, which includes brain areas such as the inferotemporal cortex (IT) [74,75].

The differential impairment of motion perception in COS can be attributed to several factors. First, the dorsal visual pathway’s neural connections and processing mechanisms may be more susceptible to disruptive effects, leading to impaired motion perception. This could be due to genetic factors, developmental abnormalities, subtle acquired brain injuries (e.g., perinatal complications), or the interaction of these factors [65,76]. Second, the impairment may be explained by a sensitivity to motion cues. Motion perception relies on detecting and interpreting subtle changes in visual stimuli, such as direction and speed. Any impairment of the sensitivity to these motion cues can result in difficulty perceiving and analyzing motion accurately [36]. Third, motion perception requires integrating spatial and temporal information to perceive coherent motion. Impairments in integrating these cues can lead to difficulty perceiving and tracking moving objects [37,53].

In contrast, form perception may be relatively less impaired due to the involvement of different brain regions and processing mechanisms. The ventral visual pathway, responsible for form perception, may be less affected by the specific impairments or dysfunctions that impact the dorsal visual pathway in COS. It is important to note that the impairment of motion perception compared to form perception may vary among individuals and can be influenced by specific underlying causes or conditions. Further research is needed to fully understand the complex mechanisms and factors contributing to these differences in visual perception impairments.

The fact that we observed marked motion perception dysfunctions in COS is consistent with the dorsal visual stream vulnerability hypothesis, claiming that developmental anomalies in motion-sensitive higher-level visual areas contribute to perceptual impairments in neurodevelopmental disorders, including autism spectrum disorders, Williams syndrome, fragile X syndrome, developmental coordination disorder, and even childhood hemiplegia, which substantially interferes with normal brain development [55,57,58,60,61,62,64,65]. The results from the present study add further new details to the literature, placing COS in the group of neurodevelopmental disorders, as mentioned earlier. Our previous research also suggests that developmental delays in motion perception are a heritability marker of schizophrenia. Namely, children of mothers with schizophrenia exhibited a less effective development of motion perception between the ages of 7–11 years relative to children with a negative family history of schizophrenia and children of mothers with bipolar disorder [71].

Why is the development of the dorsal visual system unique in childhood? Intriguingly, evidence suggests that the functions of the dorsal stream, specifically the intraparietal sulcus serving visual attention and its connections with the cerebellum, are reasonably mature in young children (4–7 years old) during visually guided action [77]. Benassi et al. (2021) recently analyzed the developmental trajectories of global motion and global form discrimination in a large sample of individuals (4–17 years of age and adults) when essential visual functions (e.g., visual acuity, stereopsis) and general cognitive development were controlled for [78]. The results revealed a marked relationship between general cognitive abilities, essential visual functions, and motion and form perception development. Interestingly, global motion perception showed an accelerated maturation compared with global form perception, which may reflect its vulnerability to genetic factors and adverse environmental circumstances. However, studies also highlighted a prolonged neural development within the dorsal pathway (age 5–6 years vs. adults), with enhanced activity in specialized regions such as the accessory visual area 3 (V3a in the superior occipital cortex, residing between lower-level (e.g., V1 and V2) and upper-tier visual cortices) and the parietal shape area (PSA) during perception tasks involving motion and structure-from-motion (coherently moving dots forming a 3D rotating object) [79]. 

Atkinson and Braddick (2020) emphasized that the crucial function in development is the emergence of cortical selectivity, the integration of local signals for providing general representations of motion, shape, and space, the building of visuomotor modules for eye movements, manual reaching, and locomotion, and the emergence of attentional systems [76]. Developmental anomalies in the dorsal cortical stream system are reflected by measures of global motion processing, visuomotor actions, and attention, suggesting that it is especially fragile in children with numerous neurodevelopmental disorders, including COS. However, future research is necessary to conclude that dorsal stream vulnerability is a reliable cognitive marker of COS.

The detection of motion perception dysfunctions in COS opens new directions in research. As a cognitive marker of neurodevelopmental deviations, it can be utilized in functional neuroimaging studies to detect specific biases in brain activation and connectivity. Moreover, genetic factors that are identified in COS (e.g., copy number variations, rare protein-truncating variants) may be more directly related to dorsal visual stream abnormalities than clinical symptoms [80].

There are a couple of limitations that should be considered in the interpretation of the results. First, the sample size was small. However, COS is a rare clinical condition (0.04% of children), limiting recruitment [3]. To deal with these disadvantages of study design, we used Bayesian statistics and conventional approaches that revealed strong evidence for motion perception impairments in COS. Second, the study was cross-sectional, which does not allow for the dynamics of motion perception development in COS to be assessed. Third, only a single test was used to measure form and motion perception. The reason for our limited test battery is that patients with COS cannot stay on task for an extended period.

## 5. Conclusions

In conclusion, we demonstrated impaired visual form and motion perception in COS, even when the general intellectual functions and socioeconomic status were controlled for. The deficits in motion perception were more severe than in form perception, which is consistent with the dorsal visual system vulnerability hypothesis of neurodevelopmental disorders. Further studies are warranted to elucidate the functional consequences of motion perception alterations in COS and to gain insight into its neuronal and genetic correlates.

## Figures and Tables

**Table 1:** Characteristics of the participants.

	Controls (*n* = 21)	Childhood-Onset Schizophrenia (*n* = 21)
Male/female	15/6	15/6
Age (years)	19.3 (4.2)	19.5 (4.8)
Parental socioeconomic status (Hollingshead)	46.8 (17.9)	47.0 (15.3)
IQ (Wechsler-IV)	89.6 (11.4)	90.3 (12.5)
Duration of illness (years)	-	9.2 (3.4)
PANSS—positive symptoms	-	19.3 (5.7)
PANSS—negative symptoms	-	21.6 (8.0)
PANSS—general symptoms	-	45.9 (13.1)
Type of antipsychotics	-	Clozapine (*n* = 17) Risperidone (*n* = 3) Olanzapine (*n* = 1)
Chlorpromazine-equivalent dose of antipsychotics (mg/day)		342.2 (119.3)

Data are mean (standard deviation), except sex distribution and type of antipsychotics. The two groups did not differ in sex distribution, age, parental socioeconomic status, and IQ (*p* > 0.5).

## Data Availability

The data are available from the authors upon request.
